# Combination Network with Multiaccess Caching

**DOI:** 10.3390/e28020220

**Published:** 2026-02-13

**Authors:** Bowen Zheng, Yifei Huang, Dianhua Wu

**Affiliations:** 1 Key Lab of Education Blockchain and Intelligent Technology, Ministry of Education, Guangxi Normal University, Guilin 541004, China; 2 School of Science, Guilin University of Aerospace Technology, Guilin 541004, China; 3 Guangxi Key Lab of Multi-Source Information Mining and Security, Guangxi Normal University, Guilin 541004, China; dhwu@gxnu.edu.cn; 4 The Center for Applied Mathematics of Guangxi, Guangxi Normal University, Guilin 541006, China

**Keywords:** coded caching, combinatorial network, multiaccess, placement delivery array

## Abstract

In the traditional (H,r,M,N) combination network, a central server storing *N* files communicates with K=(Hr) users through *H* cache-less relays. Each user has a local cache of size *M* files and is connected to a distinct subset of *r* relays. This paper studies the (H,r,L,Λ,M,N) combination network with multi-access caching, where Λ cache nodes (each of size *M* files) are available and each user can access *L* cache nodes. We show that in the regime H≥Λ and r≥L, an achievable design can be obtained via a group-wise operation, which reduces the scheme design within each group to an effective (Λ,L,L,Λ,M,N) instance. For the case Λ=H and L=r, we further propose an explicit coded caching scheme constructed via two array-based representations (a cache-node placement array and a user-retrieve array) and a derived combinatorial placement delivery array (CPDA) based on the Maddah-Ali–Niesen (MN) placement strategy. Numerical comparisons using the user-retrievable cache ratio as the evaluation metric indicate that the proposed scheme approaches the converse bound of the traditional combination network, and the performance gap diminishes as the cache ratio increases.

## 1. Introduction

With the explosive growth of mobile data traffic, caching has become an effective technique to alleviate network congestion, especially during peak hours. By pre-storing part of the content at the network edge, caching not only reduces redundant transmissions but also significantly improves the user experience. Maddah-Ali and Niesen introduced the concept of coded caching for the shared-link network in [1]. This approach combines caching and network coding to create coded multicasting opportunities among users, thereby significantly reducing the delivery load during peak traffic periods.

As described in [1], the shared-link network consists of a single server with a library of *N* equal-length files, each of size *B* bits, and *K* users, each equipped with a local cache memory of size MB bits, where 0≤M≤N. The server is connected to all users through an error-free shared link. This setup is referred to as a (K,M,N) single-input single-output (SISO) shared-link system. A coded caching scheme for such a system operates in two phases: the placement phase and the delivery phase. In the placement phase, user demands are not yet revealed, the server prefetches part of the file into the users’ caches based on the demand statistics. The main limitation in this phase is the cache memory size. In the delivery phase, each user independently requests a file from the library. Based on these demands and the cached packets, the server sends coded packets that simultaneously satisfy multiple users. The main limitation in this phase is the transmission load required to fulfill the demands. The transmission load (or simply the load) *R* represents the total normalized amount of transmitted data under the worst-case demand. The coded caching gain is defined as g=K(1−M/N)/R, where K(1−M/N) corresponds to the transmission load of a conventional uncoded caching scheme. The objective is to design a scheme that minimize *R*, or equivalently, to maximize the gain *g*.

The first coded caching scheme was proposed in [1], which is referred to as the MN scheme. The MN scheme employs an uncoded placement (If the packets are directly placed in each user’s cache, it is called uncoded placement. Otherwise it is called coded placement) and a coded delivery strategy, enabling each broadcast message from the server to simultaneously serve multiple users. Under the constraint of uncoded placement, the MN scheme achieves the optimal transmission load when K≤N [2], and for arbitrary user demands, an optimal scheme under the same constraint was proposed in [3] by eliminating redundant transmissions in the MN scheme.

However, the subpacketization of the MN scheme increases exponentially with the number of users *K*. To address the large subpacketization problem, ref. [4] introduced a combinatorial structure called the placement delivery array (PDA), which was proposed to study coded caching schemes with low subpacketization in shared-link networks. Subsequently, various coded caching schemes with lower subpacketization were proposed based on the concept of PDA, such as [4,5,6,7,8,9]. Moreover, the concept of coded caching has since been extended to a variety of network topologies, such as device-to-device (D2D) networks [10], hierarchical networks [11], multi-server linear networks [12], and combination networks [13].

Compared with the shared-link network, a more practical network setting where users communicate with the server through intermediate relays has attracted significant attention. Since analyzing relay networks with arbitrary topologies is challenging, many studies have focused on a symmetric version of this general problem, known as combination networks with end-user-caches, which was first introduced in [13]. This system consists of a single server storing *N* files, each of size *B* bits, which is connected to *H* relays (without cache memories) via *H* orthogonal links. There are K=(Hr) users, each equipped with a cache of size MB bits and connected to a unique subset of *r* relays through *r* orthogonal links. This system is referred to as an (H,r,M,N) combination network. The transmission load for each relay is defined as the maximum normalized transmission amount from the server to each relay over all relays and all possible demands. The objective is to design a coded caching scheme that minimizes both the transmission load and the subpacketization.

Extensive research efforts have been devoted to investigating the coded caching problem in combination networks [13,14,15,16,17,18,19]. In [13], two caching schemes were proposed for combination networks: one based on a routing strategy and the other employing Maximum Distance Separable (MDS) codes. In [14], several caching schemes were proposed based on the placement strategy of the MN scheme, and by leveraging the acyclic index coding converse together with the network topology, the authors derived the state-of-the-art tightest converse bound under uncoded placement. In [15], a more general combination network was investigated, where both relays and users are equipped with cache memories (when the cache size of each relay is zero, the system model in [15] is the same as the traditional combination network model). The authors proposed a coded caching scheme that partitions the combination network into *H* independent shared-link networks and applied existing achievable schemes for the shared-link network. Ref. [17] further improved the coded placement strategy in [15] by introducing an asymmetric coded placement design, which exploits the topological symmetry to generate and deliver multicast messages, as further discussed in [16].

All the aforementioned schemes have extremely high subpacketization, which leads to high implementation complexity. To address this issue, the authors in [18] introduced the combinatorial placement delivery array (CPDA), and a low-subpacketization scheme was then proposed under the constraint that *r* divides *H* (denoted by r∣H). Subsequently, ref. [19] presented improved CPDA constructions that eliminated the restriction r∣H.

Existing studies on combination networks have primarily focused on the conventional setting, where each user is equipped with an independent local cache and is connected to a fixed subset of relay nodes. In this model, caching is performed exclusively at the user side, while the relay nodes serve only as forwarding entities without storage capabilities.

**Figure 1:**
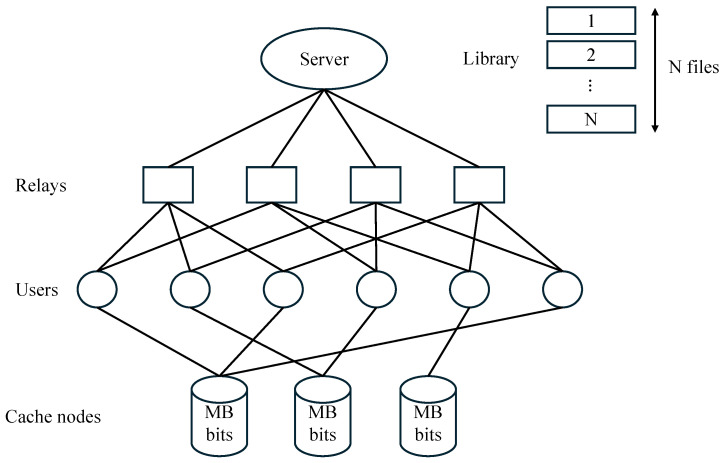
A combination network with multi-access caching with H=4 relays and Λ=3 cache nodes, where K=(42)=6 users, each connected to r=2 relays and L=1 cache node.

Motivated by emerging edge caching architectures in which users may access shared cache resources deployed at the network edge (e.g., access points or edge servers), we consider a general variant of the combination network with multi-access caching, referred to as the (H,r,L,Λ,M,N) combination network with multi-access caching. In this setting, users no longer possess individual local caches; instead, each user can access shared cache nodes deployed at the network edge. As illustrated in Figure 1, the system consists of a single server storing a library of *N* files, each of size *B* bits, which is connected to *H* cache-less relays through *H* orthogonal, error-free links. There are K=Hr cache-less users in the network, each associated with a distinct subset of *r* relay indices and connected to the corresponding relays via *r* orthogonal, error-free links. At the network edge, Λ cache nodes (also referred to as edge nodes) are deployed, each with a storage capacity of MB bits, where 0≤M≤N. Each user can access any *L* cache nodes, retrieve cached contents from these nodes without any cost, and receive server transmissions through the connected relays. The objective is to design a coded caching scheme that minimizes both the worst-case normalized transmission load over the server-to-relay links and the subpacketization.

Our main contributions are summarized as follows.

- We introduce two combinatorial structures, termed the cache-node placement array and the user-retrieve array, to characterize cache placement and packet retrievability under multi-access caching.
- For H≥Λ and r≥L, we reduce the scheme design to an effective (Λ,L,L,Λ,M,N) instance via a group-wise operation; for Λ=H and L=r, we further construct the proposed arrays and derive a CPDA based on the Maddah-Ali–Niesen (MN) placement strategy, yielding a coded caching scheme.
- We analyze the transmission load of the proposed scheme and provide numerical comparisons with the converse bound of the traditional combination network. The results show that, when evaluated with respect to the user-retrievable cache ratio, the performance gap to the converse bound diminishes as the cache ratio increases.

**Notation:** In this paper, we use the following notations. Bold capital letters and curlicue letters denote arrays and sets, respectively; |A| denotes the cardinality of a set *A*; define [a]={1,2,…,a}, and [a:b]={a,a+1,…,b}. For any array P, P(i,j) denotes the entry in the $i^{\mathrm{th}}$ row and the $j^{\mathrm{th}}$ column. We denote ([H]a)={A∣A⊆[H],|A|=a}, i.e., ([H]a) is the collection of all subsets of [H] of cardinality *a*.

## 2. Preliminaries

In this section, we first review the definition of the placement delivery array (PDA). Next, we review the traditional (H,r,M,N) combination networks and a tool used to characterize their caching schemes, namely the combinatorial placement delivery array (CPDA). Finally, we formulate the (H,r,L,Λ,M,N) combination network with multi-access caching considered in this paper.

### 2.1. Placement Delivery Array

The placement delivery array (PDA) was introduced in [4] to characterize the placement and delivery issues in shared-link networks and is defined as follows.

**Definition** **1** (PDA [4])**.** *positive integers K,F,Z and S, an F×K array Q=(Q(f,k)), where f∈[F],k∈[K], composed of a specific symbol “∗” and integers in [S], is called a (K,F,Z,S) placement delivery array (PDA) if the following conditions hold:*

*C1.*  *Each column has exactly Z stars.*
*C2.*  *Each integer in [S] occurs at least once.*
*C3.*  *For any two distinct entries $Q(f_{1},k_{1})$ and $Q(f_{2},k_{2})$, $Q\left(f_{1},k_{1}\right)=Q\left(f_{2},k_{2}\right)=s$ if only if*
  *a.*  *$f_{1}\neq f_{2},k_{1}\neq k_{2}$, i.e., they lie in distinct rows and distinct columns;*
  *b.*  *$Q\left(f_{1},k_{2}\right)=Q\left(f_{2},k_{1}\right)=*$, i.e., the corresponding 2×2 subarray formed by rows $f_{1},f_{2}$ and columns $k_{1},k_{2}$ must be one of the following form,*
    (1) $$(\begin{array}{cc} s & *\\ {}* & s \end{array})\;\;\;\;or\;\;\;\;(\begin{array}{cc} {}* & s\\ s & * \end{array}).$$

Note that, for ease of notation, the non-star entries in a PDA may sometimes be expressed using sets or vectors rather than integers.

Given a (K,F,Z,S) PDA, an *F*-division (K,M,N) coded caching scheme can be obtained by interpreting rows as subpackets and columns as users, where the symbol “∗” indicates that the corresponding packet is cached at the user, while each non-star entry (integer, set, or vector) denotes a coded multicast transmission in the delivery phase.

Moreover, a PDA is called *g*-regular if each integer occurs exactly *g* times. The MN coded caching scheme proposed by Maddah-Ali and Niesen in [1] can be represented by a PDA, which is referred to as the MN PDA. It is defined as a (Kt)×K array Q=(Q(T,k))T∈([K]t),k∈[K]

$$\begin{array}{c} Q\left(T,k\right)=\left\{\begin{array}{cc} {}*, & \mathrm{if}\;k\in T,\\ T\cup \{k\}, & \mathrm{if}\;k\notin T, \end{array}\right. \end{array}$$

and t=KM/N∈[0,K]. This means that each file is split into (Kt) packets, each labeled by a *t*-subset of [K]. In an MN PDA, the $j^{th}$ row represents the $j^{th}$ packet of every file in the library, and the $k^{th}$ column corresponds to user *k*. An entry is “∗” if the user caches that packet (i.e., the user index belongs to the subset); otherwise, the entry is an integer or a vector indicating that the corresponding packets are simultaneously delivered in a coded transmission.

**Example** **1.** *It is easy to verify that the following array Q is a *3-*(4,6,3,4) MN PDA:*

$$\begin{array}{ccc} & \begin{array}{cccc} \;\;1\;\; & \;\;2\;\; & \;\;3\;\; & \;\;4\;\; \end{array} & \\ Q= & \left(\begin{array}{cccc} {}* & * & 123 & 124\\ {}* & 123 & * & 134\\ {}* & 124 & 134 & *\\ 123 & * & * & 234\\ 124 & * & 234 & *\\ 134 & 234 & * & * \end{array}\right) & \begin{array}{c} 12\\ 13\\ 14\\ 23\\ 24\\ 34 \end{array} \end{array}.$$

*In (1), each subset is written as a string for short. For instance, the set {1,2} is written as *12*.*

### 2.2. Traditional Combination Network and Combinatorial Placement Delivery Array

In an (H,r,M,N) combination network [13], a server has access to a library of *N* files, denoted by $W=\{W_{n}|n\in \left[N\right]\}$, where each file $W_{n}$ consists of *B* independent and uniformly distributed random bits, and is connected through error-free and interference-free links to all *H* cache-less relays, say [H]. The relays are connected via error-free and interference-free links to K=(Hr) users, denoted by K={Uk∣k∈[K],Uk∈([H]r)}. Each *r*-subset $U_{k}$ of relay indices uniquely represents a user, and specifies the set of relays to which this user is connected, i.e., $\left\{h:h\in U_{k}\right\}$. The set of relays which connect to user k∈[K] is denoted by $H_{k}$. Each user has a local cache memory of size MB bits.

The system operates in two phases:

- **Placement phase:** During the off peak traffic times, the server divides each file $W_{n}$ into *F* equal-length packets, i.e., $W_{n}=\left\{W_{n,f}|f\in \left[F\right]\right\}$. The parameter *F* is referred to as the subpacketization. Each user k∈[K] directly accesses to the file library W and stores an arbitrary function thereof in its cache memory, subject to the space limitation of MB bits. For each user k∈[K], the cache contents are denoted by $Z_{k}≜\mu_{k}\left(W_{n}:n\in \left[N\right]\right)$, where $\mu_{k}$ is a placement function $\mu_{k}:F_{2}^{N\cdot E}\to F_{2}^{M\cdot E}$. The set of all cache contents is denoted by $Z≜\{Z_{k}:k\in \left[K\right]\}$.
- **Delivery phase:** During the peak traffic times, each user k∈[K] requests a file $W_{d_{k}}$ from the server, where $d_{k}\in \left[N\right]$. The demand vector is $d=\{d_{1},d_{2},\cdots ,d_{K}\}$, which is revealed to the server, the relays, and all users. For each h∈[H], the server transmits a message to relay *h*:
  $$\begin{array}{c} X_{Ser\to h}=\psi_{Ser\to h}\left(W,Z,d\right), \end{array}$$
  where $\psi_{Ser\to h}$ is an encoding function $\psi_{Ser\to h}:F_{2}^{N\cdot B}\times F_{2}^{K\cdot M\cdot B}\times {\left[N\right]}^{K}\;\to \;F_{2}^{L_{Ser\to h}}$, and $L_{Ser\to h}$ is the size of $X_{Ser\to h}$. Each relay *h* forwards $X_{Ser\to h}$ to all connected users. Each user *k* recovers its desired file $W_{d_{k}}$ from its accessible cache contents $Z_{k}$, the received signals $\left\{X_{Ser\to h}:h\in U\right\}$, and the demand vector d, i.e.,
  $$\begin{array}{c} W_{d_{k}}=\phi_{k}(\left\{X_{Ser\to h}:h\in U\right\},Z_{k},d), \end{array}$$
  where $\phi_{k}$ is a decoding function $\phi_{k}:F_{2}^{\sum_{h\in U}L_{Ser\to h}}\times F_{2}^{M\cdot B}\times {\left[N\right]}^{K}\;\to \;F_{2}^{B}$.

In such system, the worst-case number of files transmitted by each relay (also referred to as the load) over all possible requests should be minimized, which is defined as

(2) $$\begin{array}{c} R_{h}=\max_{d\in {\left[N\right]}^{K}}\{\frac{L_{Ser\to h}}{B}\}. \end{array}$$

For the coded caching problem in combination networks, the authors in [18] proposed a combinatorial structure, called the combinatorial placement delivery array (CPDA), which characterizes the placement and delivery issue in combination networks. Its definition is given as follows.

**Definition** **2** (CPDA [18])**.** *For positive integers H,r,F,Z,S, let K=(Hr). An F×K array P with alphabet [S]∪{∗} is called a (K,F,Z,S) combinatorial placement delivery array (CPDA) if it satisfies conditions C1–C3 in Definition 1 and the following conditions:*

*C4.*  *All the columns are labeled by K={Uk∣k∈[K],Uk∈([H]r)}, such that for any s∈[S], the intersection of all column labels $U_{k}$ in which s appears, denoted by $R_{s}$, is non-empty.*

Using Algorithm 1, which was proposed in [19], a (K,F,Z,S) CPDA leads to a coded caching scheme for the (H,r,M,N) combination network.
**Algorithm** **1** Caching scheme based on (K,F,Z,S) CPDA in [19]1:**procedure** Placement(P, W)2:      Split each file $W_{n}\in W$ into *F* packets, i.e., $W_{n}=\left\{W_{n,f}|f\in \left[F\right]\right\}$.3:      **for** k∈[K] **do**4:            $Z_{k}\leftarrow \left\{W_{n,f}|p_{f,U_{k}}=*,\;\forall f\in \left[F\right],U_{k}\in K,n\in \left[N\right]\right\}$5:      **end for**6:**end procedure**7:**procedure** Delivery(P,W,d)8:      **for** s∈[S] **do**9:            $X_{s}={⨁}_{P(f,k)=s,\;f\in [F],\;k\in K}W_{d_{k},f}$10:          $R_{s}={⋂}_{P(f,k)=s,\;f\in [F],\;k\in K}H_{k}=\left\{h_{s,1},h_{s,2},\ldots ,h_{s,\omega_{s}}\right\},\;1\leq \omega_{s}\leq r$11:          Divide $X_{s}$ into $\omega_{s}$ sub-packets, i.e., $X_{s}=\left\{X_{s,1},X_{s,2},\ldots ,X_{s,\omega_{s}}\right\}$12:          **for** $l\in [\omega_{s}]$ **do**13:                Server sends $X_{s,l}$ to relay $h_{s,l}$14:                Relay $h_{s,l}$ broadcasts $X_{s,l}$ to its connecting users15:          **end for**16:   **end for**17:**end procedure**

**Lemma** **1** ([19])**.** *Given a (K=(Hr),F,Z,S) CPDA and using Algorithm 1, we have an (H,r,M,N) coded caching scheme with M/N=Z/F, subpacketization less than or equal to rF, and transmission rate for each relay $R_{h}=\frac{S}{HF}$.*

Let us take the following example to further explain the connection between CPDA and Algorithm 1.

**Example** **2.** *It is easy to verify that the following array P is a (K=10,F=5,Z=2,S=10) CPDA.*

$$\begin{array}{ccc} & \begin{array}{cccccccccc} \;123\; & \;124\; & \;125\; & \;134\; & \;135\; & \;145\; & \;234\; & \;235\; & \;245\; & \;345\; \end{array} & \\ P= & \left(\begin{array}{cccccccccc} \;\;6\;\; & \;\;5\;\;\; & \;\;\;4\;\; & \;\;\;3\;\;\; & \;\;\;2\;\; & \;\;\;1\;\;\; & \;\;\;*\;\;\; & \;\;\;*\;\;\; & \;\;\;*\;\;\; & \;\;\;*\;\;\;\\ \;\;9\;\; & \;\;8\;\; & \;\;7\;\; & \;\;*\;\; & \;\;*\;\; & \;\;*\;\; & \;\;3\;\; & \;\;2\;\; & \;\;1\;\; & \;\;*\;\;\\ \;\;10\;\;\; & \;\;*\;\; & \;\;*\;\; & \;\;8\;\; & \;\;7\;\; & \;\;*\;\; & \;\;5\;\; & \;\;4\;\; & \;\;*\;\; & \;\;1\;\;\\ \;\;*\;\;\; & \;\;10\;\;\; & \;\;*\;\; & \;\;9\;\; & \;\;*\;\; & \;\;7\;\; & \;\;6\;\; & \;\;*\;\; & \;\;4\;\; & \;\;2\;\;\\ \;\;*\;\;\; & \;\;*\;\; & \;\;10\;\;\; & \;\;*\;\; & \;\;9\;\; & \;\;8\;\; & \;\;*\;\; & \;\;6\;\; & \;\;5\;\; & \;\;3\;\; \end{array}\right) & \begin{array}{c} 1\\ 2\\ 3\\ 4\\ 5 \end{array} \end{array}.$$

*Based on the above CPDA P, the following (H=5,r=3,M,N) combination network coded caching scheme can be obtained by using Algorithm 1.*

- ***Placement phase:*** *From line *2*, each file $W_{n}$ is divided into F=5 packets denoted as $W_{n}=\left\{W_{n,1},W_{n,2},W_{n,3},W_{n,4},W_{n,5}\right\}$. For any f∈[F]=[6] and Uk∈([5]3), $p_{f,U_{k}}=*$ represents that user k caches the $f^{th}$ packet of all files, then from line 4, the cached content of all users are*
$$\begin{array}{cccc} \; & Z_{\left\{1,2,3\right\}}=\left\{W_{n,4},W_{n,5}|n\in \left[N\right]\right\}, & \; & Z_{\left\{1,2,4\right\}}=\left\{W_{n,3},W_{n,5}|n\in \left[N\right]\right\},\\ \; & Z_{\left\{1,2,5\right\}}=\left\{W_{n,3},W_{n,4}|n\in \left[N\right]\right\}, & \; & Z_{\left\{1,3,4\right\}}=\left\{W_{n,2},W_{n,5}|n\in \left[N\right]\right\},\\ \; & Z_{\left\{1,3,5\right\}}=\left\{W_{n,2},W_{n,4}|n\in \left[N\right]\right\}, & \; & Z_{\left\{1,4,5\right\}}=\left\{W_{n,2},W_{n,3}|n\in \left[N\right]\right\},\\ \; & Z_{\left\{2,3,4\right\}}=\left\{W_{n,1},W_{n,5}|n\in \left[N\right]\right\}, & \; & Z_{\left\{2,3,5\right\}}=\left\{W_{n,1},W_{n,4}|n\in \left[N\right]\right\},\\ \; & Z_{\left\{2,4,5\right\}}=\left\{W_{n,1},W_{n,3}|n\in \left[N\right]\right\}, & \; & Z_{\left\{3,4,5\right\}}=\left\{W_{n,1},W_{n,2}|n\in \left[N\right]\right\}. \end{array}$$
- ***Delivery phase:*** *Assume that the demand vector is d=(1,2,…,K), i.e., user k requests file $W_{k}$ for k∈[10]. In the CPDA P, each s∈[12] corresponds to a coded transmission. For each s, by line 9 of Algorithm 1, the server constructs*
$$\begin{array}{c} X_{s}=\underset{\left(f,U_{k}\right):\;p_{f,U_{k}}=s}{⨁}W_{k,f}. \end{array}$$
  *Let $R_{s}=\left\{h_{s,1},h_{s,2},\ldots ,h_{s,\omega_{s}}\right\}$ be the relay set given in condition C4 of Definition *2* where $1\leq \omega_{s}\leq r,$. Then $X_{s}$ is divided into $\omega_{s}$ sub-packets $\left\{X_{s,1},\ldots ,X_{s,\omega_{s}}\right\}$, where the server sends $X_{s,l}$ to relay $h_{s,l}$ and relay $h_{s,l}$ forwards it to its connected users. Thus the subpacketization is at most rF. For example, when s=1, we obtain*
$$\begin{array}{c} X_{1}=W_{6,1}⊕W_{9,2}⊕W_{10,3},\;R_{1}=\left\{4,5\right\}. \end{array}$$
*So $X_{1}$ is split into $|R_{1}|=2$ sub-packets $X_{1,1}$ and $X_{1,2}$, delivered via relays *4* and *5*, respectively. User *6* can decode $W_{6,1}$ using $\left(X_{1,1},X_{1,2}\right)$ together with its cached packets $W_{9,2}$ and $W_{10,3}$. Similarly, users *9* and *10* recover $W_{9,2}$ and $W_{10,3}$. By the same argument, every user can decode its requested uncached packets. Table 1 summarizes the transmissions. In Table 1, each relay sends $4\times \frac{1}{2}$ packets, leading to the transmission rate for each relay $R_{h}=\frac{2}{5}$.*

The authors in [14], based on the cut-set approach in [13], extended the converse bound for shared-link coded caching under the constraint of uncoded cache placement to the (H,r,M,N) combination network.

**Lemma** **2** (Converse Bound [14])**.** *For the (H,r,M,N) combination network with N≥K=(Hr), under the constraint of uncoded cache placement, the converse bound $R^{*}$ satisfies that*

(3) R∗≥maxx∈[r:H](xt)−tx(t+1),

*where t=(xr)M/N∈[0:(xr)].*

### 2.3. Considered Model: Combination Network with Multiaccess Caching Model

In this subsection, we introduce an extended system model that integrates the combination network with multi-access caching, where each user is allowed to access *L* cache nodes deployed at the network edge.

Consider the (H,r,L,Λ,M,N) combination network with a multi-access coded caching system as illustrated in Figure 1, where *H* and *r* are positive integers with r≤H, Λ is the number of cache nodes, and *L* is the number of cache nodes accessible to each user. The system consists of a single server with a set of *N* equal-length files (denoted by $W=\{W_{n}|n\in \left[N\right]\}$), *H* cache-less relays (denoted by [H]), K=(Hr) users (denoted by K={Uk∣k∈[K],Uk∈([H]r)}), and Λ cache nodes (denoted by $C=\{C_{i}|i\in \left[\Lambda \right]\}$). Each cache node has a memory size of *M* files where 0≤M≤N. The server is connected to all *H* relays through error-free and interference-free links. Each user k∈[K] is uniquely represented by an *r*-subset $U_{k}$, and is connected via error-free and interference-free links to the *r* relays indexed by $U_{k}$, i.e., $\left\{h:h\in U_{k}\right\}$. Moreover, each user k∈[K] can access an arbitrary subset of *L* cache nodes and retrieve cached contents from these cache nodes without any cost. In this paper, we assume that the communication bottleneck lies on the server-to-relay links.

The system operates in two phases:

- **Placement phase:** During off-peak traffic times, each cache node $C_{i}$, where i∈[Λ], stores some packets of each file subject to the memory size *M*. Let $Z_{C_{i}}$ denote the cache contents at cache node $C_{i}$. Each user k∈[K] can retrieve the contents cached at its accessible cache nodes. Let $Z_{k}$ denote the cache contents retrievable by user *k*.
- **Delivery phase:** During peak traffic times, each user *k* requests a file $W_{d_{k}}$ from the server, where $d_{k}\in \left[N\right]$. The demand vector is $d={\left(d_{k}\right)}_{k\in [K]}$, which is revealed to the server, the relays, and all users. For each h∈[H], the server transmits a message $X_{Ser\to h}$ to relay *h*, and then relay *h* forwards $X_{Ser\to h}$ to all its connected users. Each user *k* recovers its desired file $W_{d_{k}}$ from its accessible cache contents $Z_{k}$, the received signals $\left\{X_{Ser\to h}:h\in U_{k}\right\}$, and the demand vector d.

The objective is to design a coded caching scheme that minimizes the worst-case load at each relay as defined in (2).

To describe the cache placement and packet retrievability under multi-access caching, we introduce two combinatorial structures, namely, the cache-node placement array C and the user-retrieve array U, which are defined as follows.

**Definition** **3.** *Let F denote the subpacketization, *Λ* the number of cache nodes, and K the number of users.*

- *The F×Λ cache-node placement array C consists of stars and nulls. The entry C(j,i)=∗ indicates that the j-th packet of each file, i.e., $\left\{W_{n,j}:n\in \left[N\right]\right\}$, is stored in cache node $C_{i}$.*
- *The F×K user-retrieve array U consists of stars and nulls. The entry U(j,k)=∗ indicates that the j-th packet of each file, i.e., $\left\{W_{n,j}:n\in \left[N\right]\right\}$, can be retrieved by user k from its accessible cache nodes.*

These two arrays provide a unified representation of which file packets are stored at each cache node and which packets can be retrieved by each user during the placement phase.

Note that, compared with the traditional combination network, the (H,r,L,Λ,M,N) combination network with multi-access caching differs in the placement phase, where users retrieve cached contents from shared cache nodes rather than storing content independently. For the delivery phase, we can still use a CPDA P to characterize the delivery strategy over the server–relay links. As a result, the design of a coded caching scheme for the (H,r,L,Λ,M,N) combination network with multi-access caching is equivalent to the construction of three arrays: the cache-node placement array C, the user-retrieve array U, and the CPDA P.

## 3. Main Results

### 3.1. Main Results and Performance Analysis

In this subsection, we present the main achievable results for the (H,r,L,Λ,M,N) combination network with multi-access caching. We first show that the design of an achievable scheme in the regime H≥Λ and r≥L can be reduced to studying an effective (Λ,L,L,Λ,M,N) setting. We then focus on this setting and provide a coded caching scheme.

**Theorem** **1.** *Consider the (H,r,L,Λ,M,N) combination network with multi-access caching. Assume that H≥Λ, r≥L, and $r<\frac{H}{2}$. An achievable scheme can be obtained by partitioning the users into groups of size at most (ΛL) and applying the same construction within each group. Equivalently, the scheme design for the (H,r,L,Λ,M,N) network reduces to designing a scheme for the (Λ,L,L,Λ,M,N) combination network with multi-access caching.*

**Proof.**  Let K=(Hr) be the total number of users, where each user k∈[K] is indexed by an *r*-subset Uk∈([H]r) of relays to which it is connected. Since H≥Λ and r≥L, each user belongs to at least one Λ-subset of relays that contains at least *L* of its connected relays.

For each B∈([H]Λ), define the (possibly overlapping) group of users

$$\begin{array}{c} G\left(B\right)≜\left\{\;k\in \left[K\right]:|U_{k}\cap B|\geq L\;\right\}. \end{array}$$

A user may belong to multiple groups G(B); in that case, we introduce a virtual copy of this user in each group in which it is served, which does not affect achievability (e.g., the virtual copies can be served in additional rounds via time-sharing). Moreover, |G(B)|≥(ΛL) holds for all B∈([H]Λ) when H≥Λ, r≥L, and $r<\frac{H}{2}$. If |G(B)|>(ΛL), we serve the users in G(B) in multiple rounds, each round handling an arbitrary subset of (ΛL) users from G(B) (and time-sharing across rounds). Therefore, we consider a group G(B) with |G(B)|=(ΛL).

Fix such a group G(B). We activate only the relays in B for serving the users in this group and keep all relays in [H]∖B silent during the transmissions intended for this group. For each user k∈G(B), choose an effective relay subset ${\tilde{U}}_{k}\subseteq U_{k}\cap B$ with $|{\tilde{U}}_{k}|=L$, and let only the relays in ${\tilde{U}}_{k}$ participate in forwarding the messages intended for user *k*. Such a choice is always possible because $|U_{k}\cap B|\geq L$ by the definition of G(B). Therefore, within this group, at most Λ relays are involved in delivery.

On the cache side, since each user can access an arbitrary subset of *L* cache nodes and |G(B)|=(ΛL), we can establish a one-to-one mapping from the users in G(B) to ([Λ]L) and assign distinct cache-access sets accordingly. Relabel the relays in B as [Λ]. Under the above relay activation and cache-access assignment, the group G(B) induces an effective (Λ,L,L,Λ,M,N) instance. Applying any scheme designed for (Λ,L,L,Λ,M,N) to each round and time-sharing across all rounds (and all B) yields an achievable scheme for the original (H,r,L,Λ,M,N) network. □

We now present the following example to further illustrate the above reduction.

**Example** **3.** *Consider an (H,r,L,Λ)=(5,2,1,3) combination network with multi-access caching. Users are indexed by U∈([5]2), so K=(52)=10.*

*Let B∈([5]3) be an arbitrary *Λ*-subset of relays. Without loss of generality, we consider B={1,2,3}. Define the associated user group as*

G(B)≜{U∈([5]2):|U∩B|≥1}.

*The only user disjoint from B is {4,5}, hence |G(B)|=9. Since (ΛL)=(31)=3, we select an arbitrary subset of *3* users to be served in one round, e.g.,*

$$\begin{array}{c} G_{0}\left(B\right)=\left\{\{1,4\},\{2,4\},\{3,4\}\right\}\subseteq G\left(B\right). \end{array}$$

*Any remaining users in $G\left(B\right)\setminus G_{0}\left(B\right)$ can be served in additional rounds via time-sharing. If desired, virtual users (replicas of existing users) can also be introduced so that a round uses any convenient set of three users; for instance, one may replicate users {1,5} and {2,5} and serve them together with {4,5} in a round.*

*We serve the users in $G_{0}\left(B\right)$ by activating only the relays in B and keeping relays in [5]∖B={4,5} silent. For each user $U\in G_{0}\left(B\right)$, choose an effective relay set $\tilde{U}\subseteq U\cap B$ with $|\tilde{U}|=L=1$ (e.g., $\tilde{U}=\left\{1\right\},\left\{2\right\},\left\{3\right\}$ for {1,4},{2,4},{3,4}, respectively).*

*On the cache side, there are Λ=3 cache nodes $\left\{C_{1},C_{2},C_{3}\right\}$ and each user can access L=1 cache node. Since ([Λ]L)={{1},{2},{3}} has size *3*, we establish a one-to-one mapping and assign*

$$\begin{array}{c} \left\{1,4\right\}↔\left\{C_{1}\right\},\;\left\{2,4\right\}↔\left\{C_{2}\right\},\;\left\{3,4\right\}↔\left\{C_{3}\right\}. \end{array}$$

*Relabel the relays in B as [Λ]=[3]. Under this relabeling, the above round induces an effective (Λ,L,L,Λ)=(3,1,1,3) instance.*

Motivated by Theorem 1, we henceforth focus on the (Λ,L,L,Λ,M,N) combination network with multi-access caching. In particular, we consider the setting Λ=H and L=r, under which we propose a coded caching scheme and obtain the following achievable result. The detailed proof is provided in Section 4.

**Theorem** **2.** *For any integers H, r, and $t^{\prime }$ such that $0<r,t^{\prime }<H$ and $r+t^{\prime }\leq H$, under Λ=H and L=r, there exists a coded caching scheme for the (H,r,L,Λ,M,N) combination network with multi-access caching achieving memory ratio $M/N=t^{\prime }/H$, subpacketization at most r2(Ht′), and per-relay transmission rate*

Rh=(r+t′)(Hr+t′)rH(Ht′).

To evaluate the performance of the proposed scheme, we compare the achievable per-relay transmission load $R_{h}$ in Theorem 2 with the converse bound derived in [14], i.e., Lemma 2. Compared with the traditional (H,r,M,N) combination network with end-user caches, the proposed (H,r,L,Λ,M,N) combination network imposes additional structural constraints in the placement phase, in the sense that users do not possess independent local caches and can retrieve cached content only through their connected cache nodes. Consequently, the set of feasible coded caching schemes under the proposed multi-access model is a subset of the feasible schemes considered in [14]. Therefore, the converse bound in [14], derived under uncoded placement and a fixed relay–user topology, remains a valid bound for the proposed model and is adopted as a performance benchmark.

Note that the converse bound in [14] is expressed as a function of the cache size available at each user. Under the proposed scheme, although the cache placement is characterized by the cache-node memory ratio $M/N=t^{\prime }/H$, each user can retrieve packets from multiple cache nodes through its combinatorial access pattern. As a result, the user-retrievable cache ratio is given by

**Figure 2:**
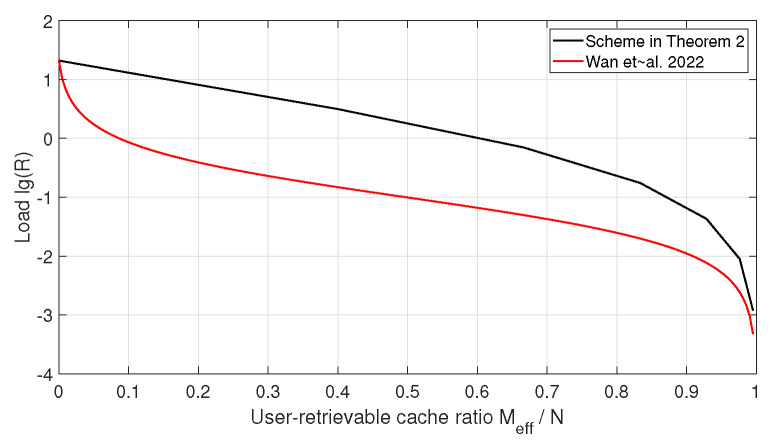
Comparison of max-link loads between the scheme in Theorem 2 and the converse bound in [14] when H=Λ=10, L=r=4, and N=210.

**Figure 3:**
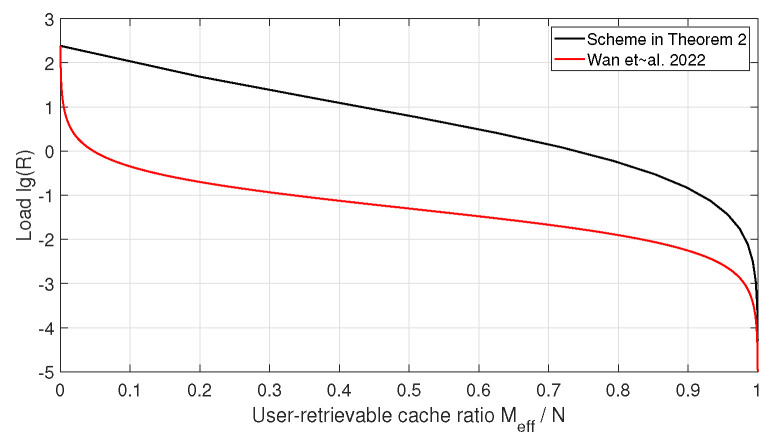
Comparison of max-link loads between the scheme in Theorem 2 and the converse bound in [14] when H=Λ=20, L=r=4, and N=4845.

MeffN=(Ht′)−(H−Lt′)(Ht′),

as defined in (6). In the following, we adopt this quantity as the performance metric for comparison. Since both the achievable per-relay transmission load in Theorem 2 and the converse bound in Lemma 2 involve binomial coefficients, obtaining a closed-form analytical comparison is, in general, intractable. Therefore, we provide numerical comparisons for several system parameters. Figure 2 and Figure 3 illustrate the comparison for H=Λ=10, L=r=4, N=210 and H=Λ=20, L=r=4, N=4845, respectively.

It can be observed that when the user-retrievable cache ratio is small, the transmission load of the proposed scheme is higher than the converse bound in [14]. As the user-retrievable cache ratio increases, the gap between the achievable load and the converse bound gradually decreases.

### 3.2. Sketch of the Proposed Scheme in Theorem 2

Consider an (H,r,L,Λ,M,N)=(4,2,2,4,3,6) combination network with multi-access caching. We specify the cache-access topology as follows. Since H=Λ=4 and r=L=2, we have K=(Hr)=(ΛL)=(42)=6. Accordingly, a one-to-one mapping can be established from ([H]r) to ([Λ]L) to determine the cache-access set of each user. For simplicity of exposition, we use Uk∈([4]2) to denote both the relay set and the cache-access set of user *k*. That is, user *k* is connected to relays $\left\{h:h\in U_{k}\right\}$ and can access cache nodes $\left\{C_{i}:i\in U_{k}\right\}$.

**Figure 4:**
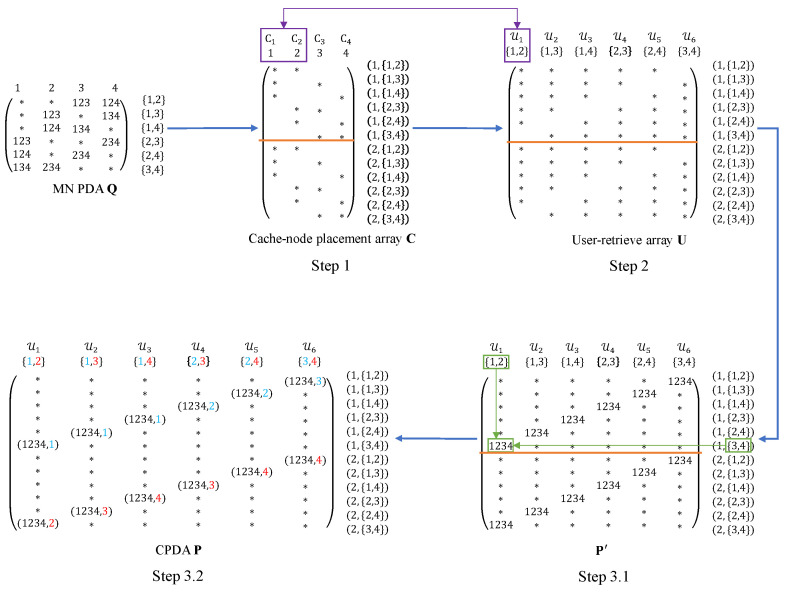
The flow diagram of constructing the cache-node placement array C, the user-retrieve array U, and the CPDA P via an MN PDA Q. The purple boxes/arrows highlight the cache-access topology of user 1 with $U_{1}=\left\{1,2\right\}$ accessing $C_{1}$ and $C_{2}$. The green boxes/arrows illustrate how the null entry at row (1,{3,4}) and column {1,2} is filled with {1,2,3,4}. The red and blue indices in the column labels indicate the second-coordinate assignment for non-∗ entries in different blocks, respectively.

Let $t^{\prime }=2$. The construction starts with the (K′=Λ,F′=(Λt′),Z′,S′)=(4,6,3,4) MN PDA Q from Example 1. Based on Q, we first construct the cache-node placement array C (for the Λ cache nodes), then derive the user-retrieve array U (for the K=(Hr)=6 users), and finally generate the corresponding CPDA P. The overall construction flow is illustrated in Figure 4.

**Remark** **1.** *When (Hr)=(ΛL), a one-to-one mapping between ([H]r) and ([Λ]L) can be established. Here, for simplicity, we use $U_{k}$ to denote both the relay set and the cache-access set of user k.*

Each file is divided into $F=rF^{\prime }=2\times 6=12$ equal-length packets, i.e.,

Wn={Wn,(j,A)|(j,A)∈[2]×([4]2)},n∈[N].

The constructing contains the following three steps:

- **Step 1. Construction of C.** We construct the cache-node placement array C by vertically replicating the placement array of the MN PDA Q (i.e., the subarray consisting of all star entries) $\left(r-1\right)$ times. As a result, the array C consists of *r* identical subarrays. For notational convenience and to avoid ambiguity, we refer to each of these subarrays as a block. According to Definition 3, a star entry in C indicates that the corresponding packet is stored at the associated cache node. As illustrated in Step 1 of Figure 4, the array C consists of r=2 identical subarrays, which are separated by an orange line. Each row of C is indexed by an ordered pair (j,A)∈[2]×([4]2), which corresponds to one packet of each file, while each column is indexed by i∈[H], representing cache node $C_{i}$. For instance, cache node $C_{1}$ stores the packets
  $$\begin{array}{c} Z_{C_{1}}=\left\{W_{n,(1,\{1,2\})},W_{n,(1,\{1,3\})},W_{n,(1,\{1,4\})},W_{n,(2,\{1,2\})},W_{n,(2,\{1,3\})},W_{n,(2,\{1,4\})}|n\in \left[N\right]\right\}. \end{array}$$
  This step ensures that the number of rows of C matches the subpacketization $F=rF^{\prime }=12$ and provides the structural basis for the subsequent construction of the CPDA.
- **Step 2. Construction of U.** We construct the user-retrieve array U according to the cache nodes accessible by each user. Specifically, each user k∈[K] is indexed by Uk∈([4]2) and can access the L=2 cache nodes indexed by $U_{k}$, i.e., $\left\{C_{i}:i\in U_{k}\right\}$. According to Definition 3, the row indices of U have the same meaning as those of C, i.e., each row corresponds to one packet of each file. Therefore, the structure of U can be directly induced by the cache-node placement array C. In particular, since the placement of star entries in C determines which packets are accessible to each user, the array U also consists of *r* identical blocks. As illustrated in Step 2 of Figure 4, the array U consists of r=2 identical blocks, which are separated by orange lines. Each row of U is indexed by an ordered pair (j,A)∈[2]×([4]2), corresponding to one packet of each file, while each column is indexed by Uk∈([4]2), corresponding to user *k*. For instance, user 1 is indexed by $U_{1}=\left\{1,2\right\}$ and can access cache nodes $C_{1}$ and $C_{2}$. So user 1 can retrieve the packets
  $$\begin{array}{cc} Z_{1}= & \{W_{n,(1,\{1,2\})},\;W_{n,(1,\{1,3\})},\;W_{n,(1,\{1,4\})},\;W_{n,(1,\{2,3\})},\;W_{n,(1,\{2,4\})},W_{n,(2,\{1,2\})},\;\\ \; & W_{n,(2,\{1,3\})},\;W_{n,(2,\{1,4\})},\;W_{n,(2,\{2,3\})},\;W_{n,(2,\{2,4\})}|n\in \left[N\right]\}. \end{array}$$
- **Step 3. Construction of P.** We construct the CPDA P based on the user-retrieve array U. Since the row and column indices of P have the same meaning as those of U, we obtain P by filling the null entries of U with appropriate symbols or vectors so that the array P satisfies all the conditions in Definition 2. The construction consists of the following two substeps.
  - **Step 3.1.** For each null entry of U indexed by row (j,A) and column $U_{k}$, we fill the entry with the union of the second coordinate of the row index and the column index, i.e., $A\cup U_{k}$, to obtain an intermediate array $P^{\prime }$. For instance, the null entry corresponding to row index (1,{3,4}) and column index {1,2} is filled with the set {1,2,3,4}. Since the second coordinate A of the row index follows the same ordering across all replicated blocks, the array $P^{\prime }$ consists of *r* identical blocks. As illustrated in Step 3.1 of Figure 4, the array $P^{\prime }$ consists of r=2 identical blocks, which are separated by an orange line. Consequently, each column of $P^{\prime }$ contains exactly Z=10 star entries, which ensures condition C1 of Definition 2. Moreover, when restricted to any single block, the array $P^{\prime }$ satisfies conditions C1–C3 of Definition 2. For instance, the first block $P^{\prime }\left(1\right)$ is given by
  $$\begin{array}{ccc} & \begin{array}{cccccc} \;\;\{1,2\}\;\; & \;\;\{1,3\}\;\; & \;\;\{1,4\}\;\; & \;\;\{2,3\} & \;\;\{2,4\} & \;\;\{3,4\}\;\; \end{array} & \\ P^{\prime }(1)= & \left(\begin{array}{cccccc} \;\;*\;\; & \;\;*\;\; & \;\;*\;\; & \;\;*\;\; & \;\;*\;\; & \;\;1234\;\;\\ \;\;*\;\; & \;\;*\;\; & \;\;*\;\; & \;\;*\;\; & \;\;1234\;\; & \;\;*\;\;\\ \;\;*\;\; & \;\;*\;\; & \;\;*\;\; & \;\;1234\;\; & \;\;*\;\; & \;\;*\;\;\\ \;\;*\;\; & \;\;*\;\; & \;\;1234\;\; & \;\;*\;\; & \;\;*\;\; & \;\;*\;\;\\ \;\;*\;\; & \;\;1234\;\; & \;\;*\;\; & \;\;*\;\; & \;\;*\;\; & \;\;*\;\;\\ \;\;1234\;\; & \;\;*\;\; & \;\;*\;\; & \;\;*\;\; & \;\;*\;\; & \;\;*\;\; \end{array}\right) & \begin{array}{c} \left(1,\{1,2\}\right)\\ \left(1,\{1,3\}\right)\\ \left(1,\{1,4\}\right)\\ \left(1,\{2,3\}\right)\\ \left(1,\{2,4\}\right)\\ \left(1,\{3,4\}\right) \end{array} \end{array}.$$
  It is not difficult to verify that $P^{\prime }\left(1\right)$ satisfies conditions C1–C3. However, since $P^{\prime }$ consists of *r* identical blocks, each column contains *r* identical non-∗ entries. As a result, the array $P^{\prime }$ does not yet satisfy conditions C3 and C4 of Definition 2.
  - **Step 3.2.** For each non-∗ entry in $P^{\prime }$ indexed by row (j,A) and column $U_{k}$, we assign the $j^{th}$ element of the column index $U_{k}$, denoted by $U_{k,j}$ (under a fixed ordering of $U_{k}$), as the second coordinate, thereby obtaining the array P. By distinguishing identical non-∗ entries appearing in different replicated blocks, this step ensures that the array P satisfies conditions C3 and C4 of Definition 2. As illustrated in Step 3.2 of Figure 4, the integers highlighted in different colors in the column labels are assigned as the second coordinates to the non-∗ entries in different blocks, respectively. Next, we verify that the array P satisfies conditions C3 and C4 of Definition 2. For instance, consider the subarray $P^{\left(1234,1\right)}$ corresponding to the vector (1234,1), i.e.,
  $$\begin{array}{ccc} & \begin{array}{ccc} \;\{1,2\}\; & \;\{1,3\}\; & \;\;\{1,4\}\; \end{array} & \\ P^{\left(1234,1\right)}= & \left(\begin{array}{ccc} \;*\; & \;*\; & \;(1234,1)\;\\ \;*\; & \;(1234,1)\; & \;*\;\\ \;(1234,1)\; & \;*\; & \;*\; \end{array}\right) & \begin{array}{c} \left(1,\{2,3\}\right)\\ \left(1,\{2,4\}\right)\\ \left(1,\{3,4\}\right) \end{array} \end{array}.$$
  We can verify that this subarray $P^{\left(1234,1\right)}$ satisfies condition C3. Moreover, the corresponding column indices satisfy {1,2}∩{1,3}∩{1,4}={1}≠∅, which ensures that condition C4 is also satisfied.

Therefore, P is a 3-(10,20,14,20) CPDA. Based on the node-placement array C, we have the memory ratio $M/N=t^{\prime }/H=\frac{2}{5}$, and based on the CPDA P, the transmission load for each relay is Rh=(r+t′)(Hr+t′)/rH(Ht′)=15.

## 4. Proof of Theorem 2

We consider the (H,r,L,Λ,M,N) combination network with multi-access caching with Λ=H and L=r. Let $t^{\prime }=\frac{\Lambda M}{N}$, where $t^{\prime }\in \left[0:\Lambda -r\right]$ is an integer. We adopt the following cache-access topology. Since Λ=H and L=r, there exists a one-to-one mapping from ([H]r) to ([Λ]L). Accordingly, each user *k* indexed by Uk∈([H]r) is assigned a unique cache-access set ψ(Uk)∈([Λ]L) and can access the cache nodes $\left\{C_{i}:i\in \psi \left(U_{k}\right)\right\}$ at no cost. For simplicity of exposition, we take ψ to be the identity mapping and write $\psi \left(U_{k}\right)=U_{k}$.

The proposed construction consists of three steps: (i) construction of the cache-node placement array C; (ii) construction of the user-retrieve array U; and (iii) construction of the CPDA P. The definitions of C and U are given in Definition 3, and the definition of P is given in Definition 2.

Each file is divided into F=r(Ht′) equal-length packets, i.e.,

Wn={Wn,(j,A)∣(j,A)∈[r]×([H]t′)},n∈[N].

### 4.1. Placement Strategy for Cache Nodes: Generation of C

Based on the sketch provided in Section 3.2, each row of C is indexed by an ordered pair (j,A)∈[r]×([H]t′), which corresponds to one packet of each file, while each column is indexed by i∈[Λ], representing cache node $C_{i}$. According to Definition 3, we define the F×H cache-node placement array C as follows:

(4) $$\begin{array}{c} C\left(\left(j,A\right),i\right)=\left\{\begin{array}{cc} {}* & \mathrm{if}\;i\in A,\\ \mathrm{null} & \mathrm{otherwise}. \end{array}\right. \end{array}$$

Accordingly, each cache node $C_{i}$ stores the following set of packets:

ZCi={Wn,(j,A)∣i∈A,n∈[N],(j,A)∈[r]×([H]t′)},i∈[H].

Hence, the memory size of each cache-node is r(H−1t′−1)N/r(Ht′)=t′N/H=M, satisfying the memory size constraint. The memory ratio is $M/N=t^{\prime }/H$.

### 4.2. Packets Retrievable to Users: Generation of U

Given the cache-node placement array C constructed above, we derive the corresponding user-retrieve array U. The rows of U are indexed by ordered pairs (j,A)∈[r]×([H]t′), corresponding to one packet of each file, and the columns are indexed by Uk∈([H]r), corresponding to user *k*.

Recall that each user k∈[K] can access the *r* cache nodes indexed by $U_{k}$, i.e., $\left\{C_{i}:i\in U_{k}\right\}$. By the construction of the cache-node placement array C, the entry C((j,A),i) is a star if and only if cache node $C_{i}$ stores the packet $W_{n,(j,A)}$, which occurs precisely when i∈A. Hence, user *k* can retrieve packet $W_{n,(j,A)}$ if and only if at least one of its accessible cache nodes stores it, i.e., if and only if $A\cap U_{k}\neq \emptyset$.

Therefore, we define the F×K user-retrieve array U as

(5) $$\begin{array}{c} U\left(\left(j,A\right),U_{k}\right)=\left\{\begin{array}{cc} {}* & \mathrm{if}\;A\cap U_{k}\neq \emptyset ,\\ \mathrm{null} & \mathrm{otherwise}. \end{array}\right. \end{array}$$

Accordingly, user *k* can retrieve the packet set

Zk={Wn,(j,A)|A∩Uk≠∅,n∈[N],(j,A)∈[r]×([H]t′)},k∈[K].

For a fixed user *k*, among all (Ht′) subsets A⊆[H] with $\left|A\right|=t^{\prime }$, the number of subsets satisfying $A\cap U_{k}=\emptyset$ is (H−rt′). Therefore, the number of retrievable subsets A is (Ht′)−(H−rt′). Since each such subset A corresponds to *r* packets indexed by (j,A)∈[r]×([H]t′), the total number of packets retrievable by user *k* is r((Ht′)−(H−rt′)). As each file is divided into F=r(Ht′) packets, the fraction of each file retrievable by user *k* through its accessible cache nodes is

(6) MeffN=r((Ht′)−(H−rt′))r(Ht′)=(Ht′)−(H−rt′)(Ht′).

### 4.3. Delivery Strategy: Generation of CPDA P

Finally, based on the sketch in Section 3.2 and the user-retrieve array U, we construct the CPDA P, which specifies the coded multicast delivery strategy. The array P has the same row and column indexing as U and is obtained by filling the null entries of U with two-dimensional vectors.

The CPDA P is generated according to the following construction.

**Construction** **1.** *For any positive integers H, r, and $t^{\prime }$ satisfying $0<r,t^{\prime }<H$ and $r+t^{\prime }\leq H$. Let K=([H]r) and F=[r]×([H]t′). Clearly, |F|=r(Ht′) and |K|=(Hr). We construct a |F|×|K| array P, where for each (j,A)∈F and $U_{k}\in K$,*

(7) $$\begin{array}{c} P\left(\left(j,A\right),U_{k}\right)=\left\{\begin{array}{cc} (A\cup U_{k},\;U_{k,j}), & \mathrm{if}\;A\cap U_{k}=\emptyset ,\\ {}*, & \mathrm{otherwise}, \end{array}\right. \end{array}$$

*where $U_{k,j}$ denotes the $j^{\mathrm{th}}$ element of $U_{k}$ under a fixed ordering of its elements.*

By construction, the CPDA P jointly characterizes the cache placement, packet retrievability, and the coded multicast delivery strategy for the considered combination network with multi-access caching with Λ=H and L=r. Therefore, we have the following result, whose proof is provided in Appendix A.

**Proposition** **1.** *For the (H,r,L,Λ,M,N) combination network with multi-access caching with Λ=H and L=r, and memory ratio $M/N=t^{\prime }/H$ where $t^{\prime }\in \left[0:H-r\right]$, the array P generated by Construction 1 is a (r+t′−1t′)−((Hr),r(Ht′),r((Ht′)−(H−rt′)),(r+t′)(Hr+t′)) CPDA, which leads to a combination network with multi-access coded caching scheme with subpacketization at most r2(Ht′) and transmission load for each relay Rh=(r+t′)(Hr+t′)rH(Ht′).*

After determining the CPDA P, the corresponding delivery procedure is as follows. From line 9 of Algorithm 1, for each integer s∈[S], the server sends the coded multicast message ${⨁}_{P\left(\left(j,A\right),U_{k}\right)=s,\;\left(j,A\right)\in F,\;U_{k}\in K}W_{d_{k},\left(j,A\right)}$ to the relays. Each relay then forwards its received message to the connected users so that each user can decode its requested packets from the multicast messages.

## 5. Conclusions

This paper studied coded caching for the (H,r,L,Λ,M,N) combination network with multi-access caching. To capture the cache placement and packet-retrievability structure, we introduced two combinatorial representations, namely the cache-node placement array and the user-retrieve array. Focusing on the case H=Λ and L=r, we proposed a coded caching scheme for the considered network. We also provided numerical comparisons using the user-retrievable cache ratio as the comparison metric, benchmarked against the converse bound of the traditional combination network. The results indicate that the proposed scheme approaches the converse bound, and the performance gap gradually diminishes as the cache ratio increases.

Future work includes extending the proposed constructions to more general, possibly non-combinatorial and heterogeneous connectivity patterns between users, relays, and cache nodes, and establishing tighter achievable and converse bounds for such irregular topologies.

## Figures and Tables

**Table 1:** Delivery strategy in Example 2.

Time Slot	Coded Signal	Relays	Transmitted Signals
1	$X_{1}=W_{6,1}⊕W_{9,2}⊕W_{10,3}$	4	$X_{1,1}$
		5	$X_{1,2}$
2	$X_{2}=W_{5,1}⊕W_{8,2}⊕W_{10,4}$	3	$X_{2,1}$
		5	$X_{2,2}$
3	$X_{3}=W_{4,1}⊕W_{7,2}⊕W_{10,5}$	3	$X_{3,1}$
		4	$X_{3,2}$
4	$X_{4}=W_{3,1}⊕W_{8,3}⊕W_{9,4}$	2	$X_{4,1}$
		5	$X_{3,1}$
5	$X_{5}=W_{2,1}⊕W_{7,3}⊕W_{9,5}$	2	$X_{5,1}$
		4	$X_{5,2}$
6	$X_{6}=W_{1,1}⊕W_{7,4}⊕W_{8,5}$	2	$X_{6,1}$
		3	$X_{6,2}$
7	$X_{7}=W_{3,2}⊕W_{5,3}⊕W_{6,4}$	1	$X_{7,1}$
		5	$X_{7,2}$
8	$X_{8}=W_{2,2}⊕W_{4,3}⊕W_{6,5}$	1	$X_{8,1}$
		4	$X_{8,2}$
9	$X_{9}=W_{1,2}⊕W_{4,4}⊕W_{5,5}$	1	$X_{9,1}$
		3	$X_{9,2}$
10	$X_{10}=W_{1,3}⊕W_{2,4}⊕W_{3,5}$	1	$X_{10,1}$
		2	$X_{10,2}$

## Data Availability

The data are available upon request from the corresponding author.

## Appendix A. Proof of Proposition 1

**Proof.** Consider the array P given in Construction 1, where we set Λ=H. For any column indexed by Uk∈([H]r), an entry $P(\left(j,A\right),U_{k})=*$ if and only if $A\cap U_{k}\neq \emptyset$. Since A ranges over all $t^{\prime }$-subsets of [H], the number of such A disjoint from $U_{k}$ is (H−rt′). Therefore, each column contains exactly

Z=r((Ht′)−(H−rt′))

stars, which is independent of the chosen column. Hence, condition C1 of Definition 2 holds. Each non-∗ entry of P is of the form $\left(A\cup U_{k},U_{k,j}\right)$, where $A\cap U_{k}=\emptyset$, $\left|A\right|=t^{\prime }$, $|U_{k}|=r$, and $U_{k,j}\in U_{k}$. Thus, each symbol can be uniquely represented as (B,h), where $B=A\cup U_{k}$ is an $\left(r+t^{\prime }\right)$-subset of [H] and h∈B. The total number of distinct symbols is

S=(r+t′)(Hr+t′).

Fix B∈([H]r+t′) and h∈B. Each occurrence of (B,h) corresponds to choosing an *r*-subset $U_{k}\subseteq B$ containing *h*, with $A=B\setminus U_{k}$ uniquely determined. The number of such $U_{k}$ is

(r+t′−1r−1)=(r+t′−1t′).

Hence, each symbol appears exactly (r+t′−1t′) times, and condition C2 of Definition 2 holds. Next, we verify condition C3. Assume that $P\left(\left(j,A\right),U_{k}\right)=P\left(\left(j^{\prime },A^{\prime }\right),U_{k^{\prime }}\right)=\left(B,h\right)$ with $\left(A,U_{k}\right)\neq \left(A^{\prime },U_{k^{\prime }}\right)$. Then $A\cap U_{k}=\emptyset$ and $A^{\prime }\cap U_{k^{\prime }}=\emptyset$. If we had both $A\cap U_{k^{\prime }}=\emptyset$ and $A^{\prime }\cap U_{k}=\emptyset$, then it would follow that $U_{k}=U_{k^{\prime }}$ and $A=A^{\prime }$, which contradicts the assumption $\left(A,U_{k}\right)\neq \left(A^{\prime },U_{k^{\prime }}\right)$. Therefore, $A\cap U_{k^{\prime }}\neq \emptyset$ and $A^{\prime }\cap U_{k}\neq \emptyset$, which implies that $P\left(\left(j,A\right),U_{k^{\prime }}\right)=P\left(\left(j^{\prime },A^{\prime }\right),U_{k}\right)=*$. Hence, condition C3 holds. Finally, we verify condition C4. Consider a symbol $\left(A\cup U_{k},U_{k,j}\right)$ appearing in column $U_{k}$. If it also appears in another column $U_{k^{\prime }}$, then necessarily $U_{k,j}\in U_{k^{\prime }}$. Thus, all columns containing the same symbol share at least one common relay, which guarantees condition C4. This completes the proof. □
